# False-Positive Effect in the Radin Double-Slit Experiment on Observer Consciousness as Determined With the Advanced Meta-Experimental Protocol

**DOI:** 10.3389/fpsyg.2019.01891

**Published:** 2019-08-22

**Authors:** Jan Walleczek, Nikolaus von Stillfried

**Affiliations:** Phenoscience Laboratories, Berlin, Germany

**Keywords:** confirmatory study design, anomalous cognition, counterfactual meta-experimentation, systematic negative control, quantum measurement problem

## Abstract

Prior work by Radin et al. (2012, 2016) reported the astonishing claim that an anomalous effect on double-slit (DS) light-interference intensity had been measured as a function of quantum-based observer consciousness. Given the radical implications, could there exist an alternative explanation, other than an anomalous consciousness effect, such as artifacts including systematic methodological error (SME)? To address this question, a conceptual replication study involving 10,000 test trials was commissioned to be performed blindly by the same investigator who had reported the original results. The commissioned study performed confirmatory and strictly predictive tests with the advanced meta-experimental protocol (AMP), including with systematic negative controls and the concept of the sham-experiment, i.e., counterfactual meta-experimentation. Whereas the replication study was unable to confirm the original results, the AMP was able to identify an unacceptably low true-negative detection rate with the sham-experiment in the absence of test subjects. The false-positive detection rate reached 50%, whereby the false-positive effect, which would be indistinguishable from the predicted true-positive effect, was significant at *p* = 0.021 (*σ* = −2.02; *N* = 1,250 test trials). The false-positive effect size was about 0.01%, which is within an-order-of-magnitude of the claimed consciousness effect (0.001%; Radin et al., 2016). The false-positive effect, which indicates the presence of significant SME in the Radin DS-experiment, suggests that skepticism should replace optimism concerning the radical claim that an anomalous quantum consciousness effect has been observed in a controlled laboratory setting.

## Introduction

Breakthroughs in science often depend on breakthroughs in scientific methodology. A scientific breakthrough might depend, for example, on a superior skill to detect the effect of an external test stimulus upon a laboratory system. The development of a measurement technique capable of detecting potentially *ultra-weak* effects – defined here as effects in the range of 0.1–0.001% and below – often represents a daunting technological challenge. In particular, in the exploration of unconventional scientific possibilities, such as in the search for anomalous mind-matter interactions related to *unproven* phenomena such as “micro-psychokinesis” (e.g., Maier et al., 2018), there could be a risk of compromising the reliability of a standard test method if one seeks to push the detection limits of the method past the limits as adopted in standard applications. Therefore, when choosing to do so, careful testing and verification of (1) the stability of the method as well as of (2) the specificity of the employed detection technology for the tested intervention should routinely accompany the pursuit of an ultra-weak-effects research program.

In recent years, the widely discussed Radin double-slit (DS) experiment has claimed scientific evidence for *anomalous* mind-matter interactions under controlled laboratory conditions (e.g., Radin et al., 2012). Specifically, the claim was reported that test subjects may interact “psycho-physically” with laser-light waves interfering in a DS-apparatus (for details, see Section “Insertion of the AMP Into the Radin DS-Experiment”). Briefly, in the Radin DS-experiment, test subjects follow precisely timed, computer-assisted instructions which serve “to direct their attention toward the double-slit apparatus or to withdraw their attention and relax” (Radin et al., 2012). This experiment suggests a remarkable technological skill which enables – apparently – the detection of miniscule, observer-dependent reductions in light-interference intensity. The effect size in percent due to attentional observer consciousness affecting light intensity – as detected with a photo-imaging device – was reported to be about 0.001% (Radin et al., 2016).

Despite the extremely small effect size, the researchers have reported that the *original* effect (Radin et al., 2012) appears to be reproducible even across different studies – at least as part of conceptual replication attempts (Radin et al., 2013, 2015, 2016). Nevertheless, given (1) the radical implications of the claim that an *anomalous consciousness effect* has been detected in a controlled laboratory setting, and (2) the fact that the anomalous effect is ultra-weak, at least by the above definition (≈0.1–0.001%), it seems reasonable to explore the following question: Could there exist an alternative explanation, other than observer consciousness, for the reported effect, such as a statistical artifact or systematic measurement bias? In other words, is there any chance that the astonishing claim based on the Radin DS-experiment has come about as a result of type-1 error, i.e., due to the misidentification of a false-positive for a true-positive effect?

A cautionary tale regarding ultra-weak-effects detection is the so-called “faster-than-light neutrino anomaly” (The OPERA collaboration et al., 2011). The neutrino anomaly was found to be reproducible over several years, but it was shown eventually to be caused by systematic measurement bias. The claimed effect size of the *anomalous neutrino effect* was on the order of 0.0001% (one part in 10,000) and the effect had achieved a high degree of statistical significance, i.e., of about six sigma. “Despite the large significance,” the researchers had warned in 2011, “of the measurement reported here and the stability of the analysis, the potentially great impact of the result motivates the continuation of our studies in order to investigate possible still unknown systematic effects that could explain the observed anomaly.” After careful, additional testing of the employed research design, a small hidden bias in the experimental set-up was finally identified, and the anomalous neutrino effect was revealed to be a false-positive effect. The identification of an alternative explanation, other than faster-than-light neutrinos, namely, a type-1 detection error, prompted the immediate retraction of the prior positive reports on the anomalous neutrino effect (The OPERA collaboration et al., 2013).

Radin and co-workers, by contrast, have presumed unlikely the possibility of a false-positive effect as an explanation of their results, and they have concluded that a genuine, i.e., true-positive, observer-consciousness effect was detected with high statistical significance (Radin et al., 2012, 2013, 2015, 2016). Naturally, if the psycho-physical influence of the intentional consciousness of a test subject on a quantum-physical process could be proven scientifically, no matter how weak this effect might be, then the implications for our view of reality, in general, and for our understanding of the foundations of quantum mechanics, in particular, would be revolutionary.

Quantum mechanics is well known to invite the possibility of many different foundational interpretations. A type of wave-function-collapse interpretation was offered as a possible explanation for the reported anomalous effect in the Radin DS-experiment (see Radin et al., 2012), whereby the particular interpretation assigns a special role to human consciousness, hence the term also of “quantum consciousness,” as part of the quantum-measurement process (e.g., von Neumann, 1932). More than 40 years ago, Hall et al. (1977) tested in the laboratory the proposal that “the reduction of the wave packet is a physical event which occurs only when there is an interaction between the physical measuring apparatus and the psyche of some observers”; however, these experiments found no evidence for any influence of the consciousness of a test subject on the targeted quantum-based process (Hall et al., 1977).

To this day, there exists no accepted scientific proof for the intentional, controlling activity of observer consciousness over quantum states or electromagnetic waves. Therefore, again, scientific claims to the contrary, as have been promoted by Radin and collaborators (Radin et al., 2012, 2013, 2015, 2016), should be viewed with reasonable caution. For example, in the case of the Radin DS-experiment, the claimed effect is derived *indirectly* by calculating the combined differences between experimental and control conditions from many 1,000 s of individual signal recordings as collected over weeks and months. In that case, the employed methodology could easily be prone to measurement bias, e.g., as a function of hidden sensitivities of the method to as-yet unknown factors or interactions, i.e., to ultra-weak influences other than those possibly manifested by observer consciousness. In particular, lacking experimental confirmation of the *specificity* of the detection method for the applied test intervention, i.e., for *intentional* observer consciousness, an investigator could easily reach false-positive conclusions.

Therefore, given the high stakes, it seems prudent to perform stringent tests for evaluating the stability over time as well as the degree of specificity of the measurement technology for detecting the intentional consciousness of a test subject in the Radin DS-experiment. For example, the specificity of the employed detection technology can be assessed quantitatively by determining the true-negative detection rate with the so-called *sham*-experiment (see Section “Sham-Experiment: Counterfactual Meta-Experimentation”). Naturally, if alternative explanations, i.e., *systematic methodological error* (SME) including statistical errors and experimental bias, could be eliminated (for details, see also Section “In Search of an Explanation for False-Positive Observer Effect Detection”), then the Radin DS-experiment might indeed represent a major advance toward scientific evidence for the psycho-physical influence of quantum-based observer consciousness upon a laboratory device.

For an explanation of what is meant by SME in the context of a concrete physical device, such as a DS-interference apparatus, the example of a biased or unbalanced roulette wheel is revealing. That is, the methodological challenges that are encountered in research involving ultra-weak-effects detection, including in the Radin DS-experiment, are similar to those faced by operators of roulette tables in a casino. The spinning wheel must be near perfectly balanced on the table in order to assure that mostly unbiased, i.e., near random, outcomes are obtained with each spin that is associated with placing a bet. That is, none of the eight octants of the wheel should indicate any higher probability than the others for being hit by the ball. However, there will invariably be a practical, operational limit in that regard for any concrete physical system such as the roulette wheel; as a result, there will *always* be a dominant octant, even if this can be revealed to the careful observer only after a large number of spins. In principle, a player could discover an imbalance in the system, e.g., an imbalance due to a one- to two-degree tilt of the wheel toward one side, and then could exploit the imbalance to place bets on the preferred octant of the wheel. As a consequence, the probability of winning will grow ever so slightly above chance, and winning would be guaranteed in the long term. In fact, cases are known when players have earned money by exploiting this loophole, i.e., the discovery of systematic and uncontrolled imbalances, and hence systematic bias, of casino roulette wheels (e.g., https://www.roulettephysics.com). In the context of scientific measurement design, this loophole will be referred to as the *SME-loophole*.

The present article describes the use of an advanced research protocol which is capable of controlling for possible detrimental effects of the SME-loophole in the Radin DS-experiment. The closing of this loophole is of particular concern in ultra-weak-effects studies for which there is no good intuition about either the size or the probability of a systematic imbalance or measurement bias as part of some experimental design. It is essential in such studies to verify *empirically* that the amount of SME is well below the level that might impede the reliable detection of the targeted effect. For quantifying the actual amount of SME, which might be intrinsic to the Radin DS-experiment, the *advanced meta-experimental protocol* (AMP; Walleczek, in preparation) was implemented in this conceptual replication attempt which was commissioned by one of the funders of the original Radin DS-experiment (Radin et al., 2012; see Section “Materials and Methods” for details).

For explanation, in the roulette-wheel paradigm, the SME could be quantified by recording hundreds, or more, of individual games on a given roulette wheel. Data could be collected until there is an amount sufficient to calculate a statistically significant difference between any one of the octants and the other seven octants. The more balanced and unbiased is the spinning wheel, the smaller will be the SME. The same is relevant for scientific measurement paradigms also: the more balanced and unbiased is a particular research design, the smaller will be the SME, as confirmed by a low false-positive detection rate; consequently, the higher will be the effective specificity of the employed detection method. Similar to the above strategy for detecting an imbalance in the roulette-wheel paradigm, the here employed AMP-based strategy can detect measurement imbalances or biases in the experimental system under investigation.

In summary, upon insertion of the AMP into the Radin DS-experiment, it was possible to determine the amount of SME – as revealed by the determination of the true-negative rate of detection – constraining the effective *specificity* of the employed measurement technology. The present analysis will conclude that the specificity of the method for detecting the potential effect of observer consciousness in the Radin DS-experiment is likely to be below that required for the reliable, i.e., artifact-free, detection of a putative effect on the order of 0.001% (Radin et al., 2016). It is questionable, therefore, at least until further stringent, pre-specified, AMP-based tests have been conducted, whether the previously claimed, anomalous effect could be a reliable indicator of a genuine, i.e., true-positive, observer-consciousness effect in the Radin DS-experiment. Next will be described the experimental methodology and the confirmatory AMP-based protocol which was implemented in this commissioned replication study of the Radin DS-experiment.

## Materials and Methods

A replication study of the Radin DS-experiment was commissioned to be performed by the same investigator who carried out also the *original* Radin DS-experiment (Radin et al., 2012). Like all previously published follow-up studies (Radin et al., 2013, 2015, 2016), this commissioned replication study did also implement a *conceptual*, and not a *direct*, replication design. Specifically, what all published follow-up studies share (Radin et al., 2013, 2015, 2016), including the present commissioned replication study also, is that a measure of the light intensity of the interference pattern of a DS-system was used as an outcome variable (Y) for detecting the potential anomalous consciousness effect.

The present work included an agreement with the Institute of Noetic Sciences (IONS) and with Dean Radin, who is the lead investigator of both the original DS-experiment as well as the commissioned replication study at the IONS^1^, to share with Phenoscience Laboratories – for independent data analysis – a digital copy of the complete, raw measurement data as well as the pre-planned statistical procedures in the form of the *original* Matlab software script, which was used for calculating the measure of DS-light intensity, i.e., the script that was used for the pre-specified, *blinded* analysis (see Section “Implementing the Confirmatory Research Design”). Phenoscience Laboratories^2^ is the research organization which is in charge (1) of overseeing the commissioned study and (2) of performing the independent analysis of the obtained results. For viewing the technical details of the employed signal processing routines, this original Matlab script, which consists of about 300 lines of code and which was used for preparing and statistically analyzing the raw measurement data for the commissioned study, can be made available upon request.

Concerning the ethical standards of using test subjects, the Institutional Review Board of the IONS approved the study and requested of each study participant to sign an informed consent form, which stated that participation in the study was voluntary, and could be discontinued at any time. Importantly, Dean Radin was responsible for selecting the participants for this replication study.

The advanced research protocol, which was used in the commissioned replication study, i.e., the AMP, was independently developed earlier by Phenoscience Laboratories investigator Jan Walleczek. The *confirmatory* AMP-based research design was implemented upon the request of Jan Walleczek, who is scientific director of the institution also that (co)funded both the original study (Radin et al., 2012) as well as the present commissioned replication study (Fetzer Franklin Fund; www.fetzer-franklin-fund.org). A full description of the AMP will be published elsewhere, including of (1) its application in weak-effects studies in general, i.e., in any branch of science and (2) its application both in the *serial* and the *parallel* format depending on the specific research needs (Walleczek, in preparation).

In summary, the experimental findings and conclusions as presented in this article are based upon the raw data, which were collected during the commissioned study using a *blinding protocol*, and which were forwarded to Phenoscience Laboratories. In the following, the employed experimental method, including the confirmatory AMP as adopted in this conceptual replication study, will be described.

### Implementing the Confirmatory Research Design

This replication study employed a pre-specified data collection method in combination with a pre-specified statistical procedure. Why this emphasis on “*pre*-specified”? In recent years, it has come to light that the reproducibility of results in many fields of science, including psychology, is more limited than was previously assumed (e.g., Munafò et al., 2017). To improve on the chance of obtaining true-positive, reproducible findings, it is widely agreed now that data analytic methods must be pre-specified and “locked-in” prior to the start of the study and to the viewing of the obtained results (e.g., Simmons et al., 2011). For example, in order to avoid the known perils of *post hoc* adjustments in the analysis of data, the methods that were adopted in this conceptual replication study in relation to (1) data collection, (2) data processing, and (3) statistical analysis, were specified *prior* to the performance of the experiments, and the blinding code was broken subsequent to performing the statistical analysis with the pre-programmed Matlab script; again, no changes to any of the employed analytic methods were allowed *after* the final results had been unblinded. This restriction is characteristic of *confirmatory* research practices, which is in contrast to the practices in *exploratory* research, where a more flexible approach is frequently adopted by the investigator (e.g., Simmons et al., 2011; Wagenmakers et al., 2012; Munafò et al., 2017). Finally, the pre-specified protocol for statistical analysis was stored on a secure server and was password protected, with the password being in possession of the funder who commissioned this confirmatory replication study.

In a subsequent article, we will demonstrate how small *post hoc* adjustments in choices regarding the setting of analytic parameters for an *exploratory* statistical analysis could be responsible for either the appearance, or the disappearance, of “positive” effects (von Stillfried and Walleczek, in preparation). Undisclosed *post hoc* adjustments have also been called “p-hacking” in the literature (e.g., Nuzzo, 2014). As will become evident, already rather small and neutral adjustments may generate greatly differing conclusions, including false-positive ones, about the gathered data in the Radin DS-experiment. By “neutral” we mean the application of adjustments in entirely identical manner across control and experimental conditions. Nevertheless, despite the neutrality of the *post hoc* adjustments, such adjustments can decide whether – or not – a statistically significant effect can be reported (von Stillfried and Walleczek, in preparation).

### Advanced Meta-Experimental Protocol

The AMP is implemented in this confirmatory replication study because – even with strictly *confirmatory* study designs (see Section “Implementing the Confirmatory Research Design”) – false-positive effects may still be generated and distort the validity of experimental findings. That is, even (1) when adopting a strictly confirmatory (pre-registered) study design and (2) when performing a technically accurate statistical analysis, then (apparent) positive results need not necessarily represent true-positive results. For example, consider the case of the secretly unbalanced roulette wheel described in Section “Introduction.” Specifically, statistically significant class-A and/or class-B errors (for explanation see Section “Sham-Experiment: Counterfactual Meta-Experimentation”) might be indistinguishable from potential true-positive effects. For the subsequent descriptions, the experimental test condition will be labeled X (e.g., X = observer consciousness), and the obtained measurement outcome will be labeled Y (e.g., Y = DS-light interference intensity).

How to verify that an observed, statistically significant X-Y correlation is – in fact – due to an effect of the tested influence X upon measurement outcome Y? How to reduce the risk that factors or influences *other* than the tested factor X did cause an observed statistical correlation? Again, hidden physical imbalances, or measurement biases, represent uncontrolled causal factors, or generally non-random (systematic) influences, which might *mimic* the (false-positive) appearance – in the case of the Radin DS-experiment – of an anomalous consciousness effect.

To be able to address these critical questions, the AMP-based research design implements two distinct sets of experiments: (1) the true-experiment and (2) the sham-experiment. Briefly, the so-called “true-experiment” is identical to the familiar (standard) experimental test involving the comparison of an experimental condition (X) to a control condition (O). By contrast, the so-called “sham-experiment” is intended to be an exact replication of the true-experiment, except for the fact that the source of conditions X and O is not present. For a detailed explanation of true- and sham-experimental conditions, consult the subsequent Sections “True-Experiment: Three Additional Control Test Categories” to “Statistical Interpretation of True- and Sham-Experiments.”

#### True-Experiment: Three Additional Control Test Categories

The “true-experiment” represents the standard experimental test involving the comparison of an experimental condition X to a control condition O. The experimental result can then be described quantitatively, for example, as X/O-ratio or in terms of the percent difference between X and O. Importantly, the AMP-based approach often involves the systematic performance of three additional control test categories in the true-experiment. These three additional test categories are included in order to test for the possibility of systematic errors related to (1) the application of condition O, (2) the application of X, and finally (3) the reversal of the test sequence of O and X (see Section “Three Additional Control Test Categories”).

#### Sham-Experiment: Counterfactual Meta-Experimentation

A powerful approach toward identifying the possible presence of *false-positive* X-Y-correlation effects is the strategy of *counterfactual meta-experimentation*. The term “counterfactual” has been adopted because – contrary to the true-experiment – in the performance of the counterfactual meta-experiment, the tested factor X has been removed from the experimental system. Specifically, in the current replication study of the Radin DS-experiment, the counterfactual measurement strategy, i.e., the so-called “sham-experiment,” seeks to verify that there is *not* present any sort of (hidden) statistical correlation effect due to (ultra-weak confounding) influences or factors – *other* than due to factor X, i.e., “anomalous observer consciousness.”

Briefly, for a given experimental methodology, what *would* have been the change in the measurement outcome (Y) if the source that delivers the experimental (X) and control (O) conditions had *not* actually been present? By mimicking – as closely as possible – the concrete experimental situation in the absence of the source of X and O, e.g., in the absence of test subjects in the Radin DS-experiment, the counterfactual meta-experiment tests *empirically* the counterfactual prediction “If X had not been present, then the statistical effect on Y would not have occurred.” Counterfactual meta-experiments, which determine the *true-negative detection rate*, i.e., the specificity for X, of an employed methodology, are done for one purpose only: to reduce the risk that a statistical X-Y correlation represents a false-positive measurement correlation (for details, see Section “Statistical Interpretation of True- and Sham-Experiments”).

Importantly, the purpose of the (counterfactual) sham-experiment is to test for the presence of (hidden) bias or systematic error as part of the *overall* scientific process which is used (1) to generate the measurement outcome variable (Y) over the *time course* of the complete study and (2) to record, calculate, and statistically analyze the measurement outcome Y. This *method*-dependent systematic error, i.e., the type of systematic error that is associated with the scientific process *itself* – independent of the application of either X or O – has been termed *class-A error*; by contrast, *condition*-dependent systematic error as a function of the biased (unbalanced) application of X- and O-conditions has been termed *class-B error* (Walleczek, in preparation).

For further explanation, consider the case of the present replication study of the Radin DS-experiment: given that the size of the claimed influence of observer consciousness on the light intensity of the DS-interference pattern was estimated by Radin et al. (2016) to be on the order of 0.001%, i.e., the claimed anomalous effect was ultra-weak by the present definition (see Section “Introduction”), an investigator *cannot* simply take for granted that *other* ultra-weak influences, i.e., those unrelated to test-subject consciousness, will be entirely absent from the employed experimental system. These possible – unrelated or unknown – influences represent ultra-weak *confounding factors*, whereby some of them might remain undetermined, and therefore could *never* be tracked over the time course of the complete study. This is a particularly grave concern in the present case because experimental confirmation is completely lacking for the *specificity* of the used detection method for the claimed ultra-weak anomalous influence X, i.e., for intentional observer consciousness.

Crucially, therefore, unless sufficiently accounted for, the effects of potentially ultra-weak, confounding factors might accumulate systematically over time to yield significant class-A error, which then might be mistaken for a true-positive effect. Since there exists no *statistical* test that could eliminate that risk, the possible influence of class-A error on a measurement outcome Y must be checked for, and eliminated, by *empirical* means. For that purpose, as was explained above, the sham-experiment performs an experiment on the performance of the experimental method itself (in the absence of the source of X and O); hence, the term “*meta*-experimental protocol.”

Finally, in the AMP, the true- and sham-experimental tests are performed in a manner such that the (counterfactual) sham-experiment *systematically* reproduces the relative *temporal positioning* of each randomly selected test category from the true-experiment for the duration of the complete study. Importantly, since the investigator is blind to the fact of whether – prior to completing the pre-planned statistical analysis – the obtained results belong to either the true- or the sham-experimental series, an unbiased, *quantitative* evaluation is possible of the reliability of the used methodology. See Section “Statistical Interpretation of True- and Sham-Experiments” regarding the statistical interpretation of true- and sham-experimental results.

#### Three Additional Control Test Categories

As was already noted in Section “True-Experiment: Three Additional Control Test Categories,” the AMP-based true-experimental series, besides the *standard* experiment, which tests the standard null hypothesis (H_0_) referred to in the AMP-based design as H_0_-true-X/O, includes the systematic performance of three further experimental categories which test for the possibility of systematic errors in association with (1) the application of the standard control condition O, (2) the application of the experimental intervention X, and finally (3) the reversal of the sequence of control condition O and experimental intervention condition X.

##### Systematic Negative Control

**Figure 1:**
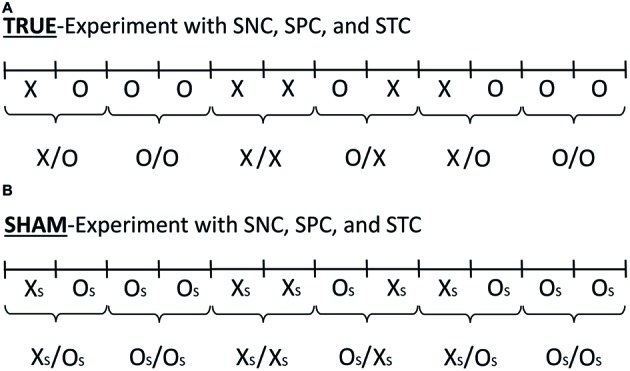
The AMP-based research design involves the true-experiment **(A)** and the sham-experiment **(B)**. The figure illustrates the *serial* AMP-8 research design (Walleczek, in preparation). The concepts of (1) standard experiment (X/O), (2) systematic negative control (SNC; O/O), (3) systematic positive control (SPC; X/X), and (4) systematic time-reversed control (STC; O/X), in the true-experiment **(A)** as well as the accompanying sham-experiment **(B)** are shown. For an explanation of the use of the SNC (O/O), SPC (X/X), and STC (O/X) test trials, see Section “Three Additional Control Test Categories.” Illustrated in **(A)** is one *possible* sequence of six successive test trials, where X and O stand for intervention and control conditions, respectively. The serial progression in time is from left to right, whereby the sham-experiment is done either before or after the true-experiment in the serial AMP-format. The sham-experiment is illustrated in **(B)** and the sham-intervention and control conditions are labeled X_S_ and O_S_, respectively. In the present work, the sham-experiment is done in the absence of any test subjects, as is indicated by the labels X_S_ and O_S_ in **(B)** in place of the labels X and O in **(A)**. A total of 10,000 experimental trials (*N* = 1,250 test trials for each of the eight pre-specified test categories in the AMP-8 format) are performed as part of the commissioned replication study.

What is a “negative” control? The term “negative control” refers to an additional control step which tests for the reliability of the control condition itself. Negative-control tests can be added – in a systematic manner – if one wishes to quantitatively assess potential systematic (class-B) errors which might be associated with the performance of the standard control condition and as used in the true-experiment throughout the complete time course of the total experimental series. The concept of the *systematic negative control* (SNC), which implements the standard negative control condition systematically, was already introduced earlier (Walleczek et al., 1999). The AMP as employed in the present study was developed as a *meta-experimental* extension of the original SNC concept (Walleczek et al., 1999; Walleczek, in preparation). The term “negative” stems from the fact that the tested intervention is – of course – absent when it would normally be present. For explanation, again, the standard experiment compares an experimental condition X with a control condition O. This standard experiment is referred to in the AMP as the true experiment “X/O” (Figure 1A). In negative-control experiments, by contrast, the condition that would normally present the true-intervention X is replaced by the control condition O. This negative-control experiment is represented in the AMP as the true-experiment “O/O” (Figure 1A). Importantly, note that the purpose of the SNC-experiment (O/O; Figure 1A), which detects *class-B* error only, fundamentally differs from that of the sham-experiment (Figure 1B), which detects *class-A* error only (for explanation see Section “Sham-Experiment: Counterfactual Meta-Experimentation”).

The performance of SNCs (i.e., systematic O/O-experiments) can confirm or disconfirm whether the standard control condition O is reliable throughout the time course of the complete study. For example, if – upon the analysis of the systematic O/O-experiment – a systematic difference between two (paired) O-conditions is observed, then this suggests the possible presence of SME in the form of class-B error (see Section “Sham-Experiment: Counterfactual Meta-Experimentation”). The null hypothesis which is tested by the SNC-experiment is referred to as H_0_-true-O/O (see Section “Reproducing the Test Categories in the Sham-Experiment”) and it holds that significant class-B error is absent as a function of *systematic* imbalances in association with the repeated application of the standard control (O) in the true-experiment.

##### Systematic Positive Control

What is a “positive control” in the context of the AMP-based strategy? The systematic positive control (SPC) experiment (X/X) allows for the quantification of SME in association with the (sequential) application of the tested true-intervention X (Figure 1A). The performance of SPCs (i.e., systematic X/X-experiments) can confirm or disconfirm whether the repeated application of the X-condition in the serial AMP format is inherently reliable. For example, the SPC-experiment might reveal that the second application of X could have either (1) a systematically stronger effect or (2) a systematically weaker effect on the measurement outcome variable (Y) than the first application of X in the test pair X/X. In short, if a significant difference between two consecutive X-conditions is observed, then this suggests the possible presence of SME in the form of class-B error (see Section “Sham-Experiment: Counterfactual Meta-Experimentation”). The null hypothesis which is tested by the SPC experiment is referred to as H_0_-true-X/X (see Section “Reproducing the Test Categories in the Sham-Experiment”), and it holds that significant class-B error is absent as a function of systematic imbalances in association with the repeated application of test stimulus X.

##### Systematic Time-Reversed Control

What are “time-reversed control” test sequences in the AMP? This third additional test category applies the control condition (O) *before* the experimental condition (X) in the pair that makes up a single experimental unit (O/X) in the serial AMP format. That is, the performance of STCs, i.e., systematic O/X-experiments, allows for the quantification of SME in the form of class-B error in association with the time-reversal of X and O conditions (see Section “Sham-Experiment: Counterfactual Meta-Experimentation”). The null hypothesis which is tested by the STC-experiment is referred to as H_0_-true-O/X (see Section “Reproducing the Test Categories in the Sham-Experiment”) and it holds that significant class-B error is absent in association with the time-reversed application of control condition O and true-intervention X; class-B error in association with the STC-experiment would be indicated if the observed effect in test category O/X is either significantly greater or smaller than, or in the *same* direction as, the observed effect in the standard true-experiment X/O (Figure 1A).

#### Reproducing the Test Categories in the Sham-Experiment

The sequence of the above-described control test categories as part of the AMP-based design (i.e., SNC, SPC, and STC) is reproduced in the sham-experiment also (Figure 1B). Importantly, the sham-experiment records the experimental data in the exact *temporal format* as given by the randomly generated sequence of individual test pairs in the true-experiment (i.e., the categories labeled X/O, O/O, X/X, and O/X). In the sham-experiment, the corresponding test categories are labeled X_S_/O_S_, O_S_/O_S_, X_S_/X_S_, and O_S_/X_S_, to indicate the absence of the source of the conditions X and O, i.e., of the test subject in the Radin DS-experiment, whereby (s) marks the *sham*-categories (compare Figures 1A,B). See Table 1 for an overview of the eight tested null hypotheses and for an explanation of the specific experimental conditions that are referred to by the different identifier labels in the commissioned replication study of the Radin DS-experiment.

**Table 1:** Overview of the identifier labels in relation to the tested experimental conditions as employed in the commissioned replication study and of the eight tested null hypotheses as part of the confirmatory AMP-based research design (compare Figure 1).

Label	Test condition	Null H = hypothesis (H_0_)
X	“Concentrate”	H_0_-true-X/O, H_0_-true-O/O H_0_-true-X/X, H_0_-true-O/X
O	“Relax”	
X_S_	No test subject	H_0_-sham-X_S_/O_S_, H_0_-sham-O_S_/O_S_ H_0_-sham-X_S_/X_S_, H_0_-sham-O_S_/X_S_
O_S_	No test subject	

*The labels X and O identify the presence of two states of consciousness, each of which is presumed to have a quantitatively different influence upon light-interference intensity (Y) in the Radin DS-experiment. The label X represents the specific state of test-subject consciousness that is associated with the instruction “concentrate,” whereas O represents the state of consciousness that is associated with the instruction “relax” in relation to the DS-apparatus (see Radin et al., 2012). Simply put, the working hypothesis of the Radin DS-experiment holds that the conscious state “relax” (O) has a markedly weaker, or opposite, “psycho-physical” influence on light-interference intensity than the conscious state “concentrate” (X). For example, the null hypothesis H_0_-true-X/O, i.e., the specific null hypothesis that is associated with test category X/O, predicts the absence of a difference between the test conditions X and O (see the right column). For the sham-experiment, the labels X_S_ and O_S_ represent the absence of either of the two states of consciousness, or of any other state of consciousness, in association with the test conditions X_S_ and O_S_ (see the lower half of the table). For example, the null hypothesis H_0_-sham-X_S_/O_S_, i.e., the specific null hypothesis that is associated with sham-test category X_S_/O_S_, predicts the absence of a difference – as a function of timing-dependent class-A error – between sham-test conditions X_S_ and O_S_ (see the right column). See the main text for details*.

Critically, in an ideal and artifact-free scenario, each of the four separate test categories in the sham-experiment (X_S_/O_S_, O_S_/O_S_, X_S_/X_S_, and O_S_/X_S_; see Figure 1B) should be incapable of rejecting their respective H_0_-sham (for the four respective null hypotheses see Table 1). That is, the four sham-test categories should perform near-identically, unless there exists a systematic imbalance (class-A error) for one or more of the test categories relative to the position of other test categories in the *time line* of the experiment. Consequently, the purpose of the four distinct null hypotheses for the *sham*-experiment listed in Table 1 is to test for the absence of class-A error in regards to the *relative* temporal positioning of the four *true*-experimental test categories X/O, O/O, X/X, and O/X, in the time line which generates and records the physical measurement outcome Y for the total experiment from start to finish (compare Figures 1A,B); again, each sham-test category should – ideally – produce null results only, because any differences between conditions X_S_ and O_S_ in the sham-experiment might only be due to their respective positions in time, i.e., due to timing-dependent class-A error (see Section “Sham-Experiment: Counterfactual Meta-Experimentation”), but not due to some “psycho-physical influence,” because no test subjects are ever present in the sham-experiment (see Table 1).

Why to reproduce precisely the *temporal sequence* of the randomly generated test categories from the true-experiment in the sham-experiment? The reason is the performance of pre-planned tests comparing the *predicted* results for the true-experiment against the *actual* results for the sham-experiment, both (1) for the random assignment of each test category and (2) for the exact temporal position of each test category in the experimental sequence in relation to all other test categories (see Section “Sham-Experiment: Counterfactual Meta-Experimentation”). Importantly, for that purpose, like the true-experiment, the sham-experiment employs fully dedicated, *non*-overlapping data sets *only* to test each separate null hypothesis, i.e., H_0_-sham-X_S_/O_S_, H_0_-sham-O_S_/O_S_, H_0_-sham-X_S_/X_S_, and H_0_-sham-O_S_/X_S_ (Table 1).

**Figure 2:**
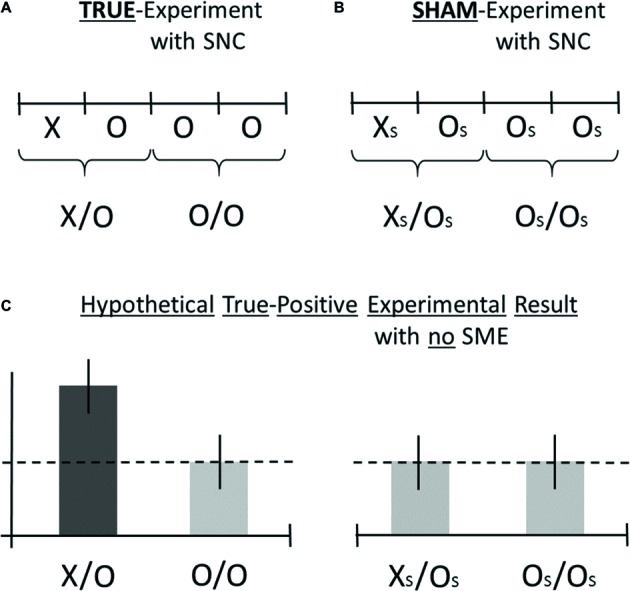
Hypothetical AMP-based measurement results are illustrated for the ideal scenario that a true-positive effect is observed in the test category X/O (dark bar), whereas the absence of false-positive effects is confirmed (bright bars) as part of some *generic* research design. The figure illustrates the AMP-4 experimental design (Walleczek, in preparation). The form of the standard, true-experiment is visualized in **(A)**, where the true-intervention is marked X, and the accompanying standard control is marked O. The formal structure of the sham-experiment is visualized in **(B)**, where the sham-intervention is marked X_S_, and the accompanying control is marked O_S_. For an explanation of the purpose of the sham-experiment, see Section “Sham-Experiment: Counterfactual Meta-Experimentation.” For demonstration purposes only, examples of hypothetical (artifact-free) findings are shown in **(C)** for the true-experimental categories X/O and O/O **(A)** as well as for the sham-experimental categories X_S_/O_S_ and O_S_/O_S_ **(B)**. In this *hypothetical* scenario, the true-positive finding represents an effect *increase* by X (dark bar) which is not challenged by systematic methodological error or SME, either in the form of class-A error or in the form of class-B error (see Section “Sham-Experiment: Counterfactual Meta-Experimentation”), as is indicated by the null results for test categories O/O, X_S_/O_S_, and O_S_/O_S_ in **(C)**. For an explanation of the SNC-experiment, see Section “Three Additional Control Test Categories.” The *dashed* line in **(C)** represents the null-effect line. For details, see main text.

Next, an ideal scenario of possible measurement outcomes for the true- and the sham-experiment is illustrated in Figure 2. Consider the *hypothetical* test result for the category X/O as shown by the dark bar in Figure 2C. How to ascertain whether the apparently positive result is either *true*-positive or *false*-positive? Or, in the opposite, hypothetical scenario (not shown), if there was not observed a positive test result but a negative one instead, then how to ascertain whether a negative result is either *true*-negative or *false-*negative? Importantly, the adoption of the confirmatory AMP-based research design can assist in answering the above questions by providing novel experimental information, i.e., information about the possible presence of class-A and/or class-B error, which is not available with standard research designs (for a detailed explanation, see the legend to Figure 2).

#### Statistical Interpretation of True- and Sham-Experiments

While a valid, successful detection of the potential effects of a test stimulus (X) on some experimental variable (Y) in the true-experiment depends strictly, of course, on X having an effect on Y, the reverse is *not* true: the capacity of the sham-experiment to help determine whether the employed method is valid – or not – need not depend on any effect, should it in fact exist, of X upon Y in the true-experiment (see Section “Sham-Experiment: Counterfactual Meta-Experimentation”). This asymmetry between what is measured by the true-experiment (see Figure 1A) *versus* what is measured by the sham-experiment (see Figure 1B), must be kept in mind – obviously – when viewing the two data sets (true vs. sham) in the context of statistical data interpretation. Importantly, again, the sham- and the true-experiment each one tests for four distinct null hypotheses in the Radin DS-experiment (see Table 1). For example, the X/O-test category alone (Figure 1A) could – in principle – reveal test results that are false-positive, true-positive, false-negative, or true-negative. By contrast, for each corresponding test category in the sham-experiment (e.g., X_S_/O_S_; Figure 1B), it is 100% certain that neither a false-negative (type-2 error) nor a true-positive test result could be measured (e.g., compare Figures 2B,C). Significantly, for the sham-experiment, the statistical probability is *known* to be zero that a true-positive effect could be observed, whereas for the true-experiment that probability is *unknown* in the Radin DS-experiment (e.g., there is no known positive control for the conscious state “concentrate,” X, other than that state itself). For an overview of the four possible interpretations of test results, see Table 2.

**Table 2:** The possible interpretations of test results in the Radin DS-experiment when they appear to be (1) positive or (2) negative: true-positive, true-negative, false-positive (type-1 error), and false-negative (type-2 error).

Test result	Effect of X on Y Yes	Effect of X on Y No	Statistical measure
Positive	True-positive	False-positive	Specificity
Negative	False-negative	True-negative	Sensitivity

*The sham-experiment seeks to determine the specificity of the detection process with measurement outcome Y for the test intervention X (e.g., observer consciousness), i.e., the true-negative rate of detection in association with the overall research design. Obviously, the higher is the true-negative rate, the lower is the false-positive rate of detection (see the upper row labeled “Positive” in the table). Put differently, a true-negative result in the sham-experiment suggests the reliability of the method, i.e., its insensitivity to factors other than the tested intervention X; conversely, a false-positive result suggests the non-reliability of the method, i.e., its non-specificity for X. See the main text for more explanations*.

In the language of statistical measurement theory, the sham-experiment in the AMP-based research design is primarily implemented for the purpose of determining the true-negative rate of detection, i.e., for determining the degree of specificity of the employed detection technology (compare Table 2). Again, the higher is the specificity of the detection process, the lower will be the frequency of false-positive detection events for the relevant test categories, e.g., for X/O and O/X (see Figure 1). Put differently, the purpose of the sham-experiment is to reduce the possibility of type-1 error as a function of experimental bias or SME in the form of class-A error (see Section “Sham-Experiment: Counterfactual Meta-Experimentation”). That is, the measurement process *itself* – in the absence of the application of either X or O – might be incapable of achieving an acceptable true-negative rate of detection, i.e., it might produce results rejecting either H_0_-sham-X_S_/O_S_ or H_0_-sham-O_S_/X_S_, which would indicate the possibility of significant class-A error in association with test categories X/O or O/X (compare Table 1).

Most importantly, a false-positive effect occurring in the true-experiment might be indistinguishable in appearance from a true-positive effect if the false-positive effect occurred in the relevant, pre-defined test category, i.e., either X/O or O/X (compare Figure 1A). Specifically, it would be impossible to tell apart a false-positive finding from a true-positive one if the false-positive effect occurred both (1) in the *predicted* temporal position of the test category in the time line of the experiment and (2) in the *predicted* effect direction, e.g., in the present Radin DS-experiment as a decrease (X/O), or an increase (O/X), respectively, in DS-light-interference intensity.

Regarding the statistical evaluation of each of the eight tested null hypotheses (see Table 1), the following explanation should be emphasized to avoid any misunderstanding: the AMP-based true- and sham-experiments employ fully dedicated, non-overlapping data sets *exclusively* to test each separate null hypothesis, i.e., H_0_-true-X/O, H_0_-true-O/O, H_0_-true-X/X, H_0_-true-O/X, H_0_-sham-X_S_/O_S_, H_0_-sham-O_S_/O_S_, H_0_-sham-X_S_/X_S_, and H_0_-sham-O_S_/X_S_ (see Figure 1 and Table 1). Therefore, since (1) neither are used multiple, or overlapping, data sets in the test of one specific null hypothesis and (2) nor are multiple null hypotheses tested using one and the same, or an overlapping, data set, calculating any type of correction for multiple comparison testing, e.g., in the form of a Bonferroni correction, would be in error. That is, for this strictly predictive study design, no multiple comparisons that would necessitate a statistical (Bonferroni) correction were programmed in the Matlab script that was used to perform the pre-planned, blinded statistical analysis (see Section “Materials and Methods”).

In summary, a stable and reliable experimental method for testing the effects of an intervention condition X should – ideally – manifest both (1) *high sensitivity* for X and (2) *high specificity* for X. Again, the statistical measure called “sensitivity” addresses the concern of misidentifying false-negatives for true-negatives (type-2 error); the more sensitive – in statistical terms – a test method is, the less false-negative results it produces (see Table 2). By contrast, the statistical measure called “specificity” quantifies the risk of misidentifying false-positives for true-positives (type-1 error); the more specific – in statistical terms – a test method is, the less false-positive results it produces (see Table 2). The additional performance of the sham-experiment, which tests for the specificity of the used methodology, can assist greatly in reducing the possibility of false-positive conclusions by quantifying the true-negative rate of detection.

### Insertion of the Advanced Meta-Experimental Protocol Into the Radin DS-Experiment

**Figure 3:**
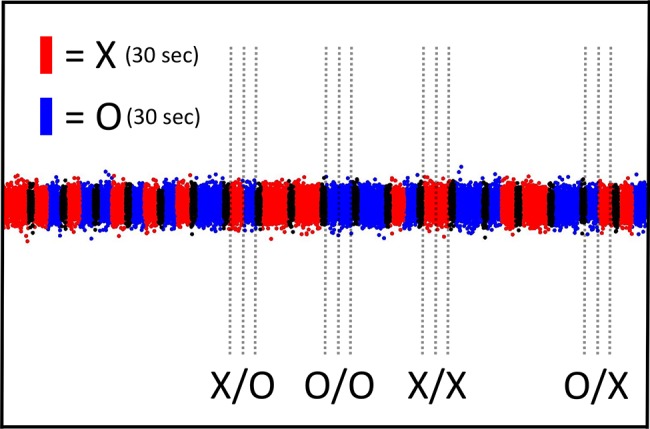
Illustration of a typical true-experiment when the test subject is present during the whole 20-min session. A segment of the real-time recording of DS-light-interference intensity by the photo-imaging camera is shown in terms of the primary outcome variable Y as defined in the pre-specified Matlab software script (for details, see Section “Materials and Methods”). The sequence of test categories or trials, i.e., X/O, O/O, X/X, and O/X, is generated randomly and is presented to the test subject in the form of computer-assisted instructions. The four possible test trials to be performed by the subject are marked in the Figure (X/O, O/O, X/X, and O/X; see also Section “Three Additional Control Test Categories”); each of the four test categories is repeated randomly five times during the 20-min session, yielding a total of 20 test trials per 20-min session. The 30-s epoch marked by the label O represents the control, i.e., “relax,” test condition. The test condition “concentrate” is represented by the 30-s epoch that is marked by the label X. After the completion of each individual test trial (i.e., 2 × 30-s epochs), a 10-s delay was added before the start of the next trial (see the short black sectors). Importantly, either before or after the 20-min true-experiment, the *paired* 20-min sham-experiment is performed. For more details see the main text in Section “Insertion of the AMP Into the Radin DS-Experiment.” For more information about the “relax” (O) and “concentrate” (X) test conditions, see the description in the legend to Table 1.

The effective use of SNC-, SPC-, and STC-experiments (see Section “Three Additional Control Test Categories”) is explained next for the concrete case of the Radin DS-experiment. One experimental session, involving one test subject, is represented by a 20-min, real-time recording of DS-light-interference intensity upon which is imposed the sequence of randomly assigned test trials X/O, O/O, X/X, and O/X (e.g., Figure 1A). The single 20-min experimental session consists of 20 individual test trials. The epochs marked “X” represent the 30-s intervals when, according to Radin et al. (2012), the test subject is instructed to affect “psycho-physically” the experimental outcome, i.e., the so-called “attention-toward,” or “concentrate,” condition (see sector X in Figure 3). By contrast, the epochs marked “O” represent the *standard* control condition, e.g., the 30-s intervals when the test subject is instructed to *not* focus on the experimental outcome, i.e., the so-called “attention-away,” or “relax,” condition (see sector O in Figure 3). For explanations of “relax” and “concentrate” conditions, see Table 1.

The sham-experiment (not shown) records the identical sequence of randomly generated trials, i.e., those that are *systematically paired* with the true-experimental session, whereby the four possible test trials of the sham-experiment are marked X_S_/O_S_, O_S_/O_S_, X_S_/X_S_, and O_S_/X_S_, to indicate the absence of the test subject (compare Figures 1A,B; see Section “Sham-Experiment: Counterfactual Meta-Experimentation”). The 20-min sham-experiment is performed either before or after the 20-min true-experiment. For more technical details regarding (1) the specifications of the used DS-apparatus, (2) the performance of the real-time recordings, and (3) the general procedures, involving test subjects, consult the original report (Radin et al., 2012).

In all, 250 sessions were performed in the presence of test subjects (true-experiment), and 250 sessions in the absence of test subjects (sham-experiment), as part of the commissioned replication study, whereby a single test session consisted of 20 separate test trials (see Figure 3). Together, the collected raw data represent a total of 10,000 individual trials, e.g., X/O, X/X, O/X, O/O, X_S_/O_S_, X_S_/X_S_, O_S_/X_S_, O_S_/O_S_, and so on, over the time course of several months. Crucially, again, for the confirmatory AMP-based strategy employed here, the exclusive purpose of six of the eight tested null hypotheses, whether they were tested in the presence (O/O and X/X) or in the absence (X_S_/O_S_, X_S_/X_S_, O_S_/X_S_, and O_S_/O_S_) of test subjects, was to reject the alternative hypothesis that SME in the form of either class-A or class-B error could be the (false-positive) cause of the reported anomalous effect in the Radin DS-experiment (compare Table 1); two of the eight tested null hypotheses, i.e., H_0_-true-X/O and H_0_-true-O/X (Table 1), were performed in the search for a true-positive effect, i.e., in an attempt to reject the null hypothesis of the original study in the presence of test subjects (Radin et al., 2012).

## Results

As was explained in Section “Materials and Methods,” a digital copy of the blindly collected raw data covering the 10,000 test trials and the original Matlab script was handed to Phenoscience Laboratories for independent data analysis and interpretation. The results described next are based on these data and the Matlab script for the statistical analysis. First in the results section will be presented the comparison of the *actual* results for the sham- and the true-experiments with the general experimental *prediction* for the true-experiment (see Sections “Prediction Versus Actual Result in the True-Experiment” and “Prediction Versus Actual Result in the Sham-Experiment”). Subsequently, an exploratory estimate of the effect size in percent, which is associated with the identified false-positive effect, will be presented in Section “Percent Effect Size of the False-Positive Observer Effect.”

### Prediction Versus Actual Result in the True-Experiment

**Figure 4:**
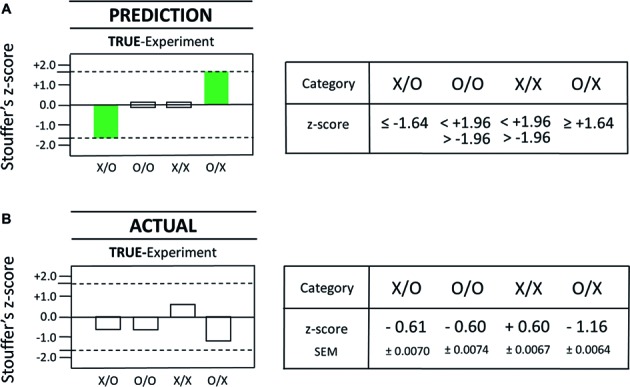
Comparison of the actual results from the true-experiment with the pre-specified outcome predictions for the true-experiment. The statistical *prediction* for test categories X/O, O/O, X/X, and O/X is displayed as a graphical (left) and a numerical (right) representation in **(A)**. The *actual* statistical results that were obtained with the true-experiment are displayed as graphical (left) and numerical (right) representations in **(B)**. Specifically, the results of the commissioned replication study are shown for the four different AMP-based test categories in the true-experiment (with test subjects) as described by Stouffer’s *z*-scores as in the original Radin DS-experiment (Radin et al., 2012). In the present case, each Stouffer *z*-score summarizes 1,250 *z*-scores calculated for each test pair which comprised a given test category. The *dashed* lines represent the magnitude of the *z*-score at ±1.64 which is the cutoff for statistical significance (one-tailed). Since this analysis was built upon a nonparametric bootstrap method involving a 300-loop random permutation process, the outcome of the pre-specified statistical analysis will vary slightly each time it is conducted. In order to generate a reliable value, we performed 100 repetitions of the pre-specified analysis and calculated means and standard errors of these means (SEM) for each category. The number of individual test trials is identical for each test category, i.e., *N* = 1,250 for X/O, O/O, X/X, and O/X, yielding a total of 5,000 test trials for the true-experiment.

A true-positive result is an experimental result as a function of the influence of the tested factor X (e.g., conscious state “concentrate”) on the measurement outcome Y (e.g., DS-light intensity). Given the strictly confirmatory study design for this conceptual replication attempt, the actual results from true- and sham-experiments are to be compared against the specific prediction of a true-positive observer effect (see Section “Statistical Interpretation of True- and Sham-Experiments”), a prediction which was, of course, specified *prior* to the performance of the actual study (see Section “Implementing the Confirmatory Research Design”). In particular, predicted was the appearance of two statistically significant, true-positive effects in the test categories X/O and O/X, whereby (1) one effect must be in the direction opposite of the other and (2) the effect direction in the X/O-test category was predicted to be a decrease. Importantly, *not* predicted were true-positive results for test categories O/O and X/X; neither could any positive findings in the X/O- and O/X-test categories, which are *opposite* to the predicted effect direction, be identified as true-positive results. In short, while eight different, statistically significant, measurement outcomes are – in principle – possible in the true-experiment, only two of these eight possible outcomes – in view of the pre-specified predictions – could represent true-positive effects as a function of X affecting Y (see the full bars in Figure 4A). The *dashed* lines in Figure 4 represent the magnitude of the *z*-score at ±1.64, which is the employed one-tailed cutoff for statistical significance. Again, for the other two test categories in the true-experiment, i.e., O/O (SNC) and X/X (SPC), the observation of null effects only was predicted (see the open bars in Figure 4A), whereby a two-tailed cutoff at *z* = ±1.96 was chosen for SNC- and SPC-test categories O/O and X/X, respectively (Figure 4A). Again, the working hypothesis of the confirmatory study predicted that true-positive effects of opposite effect direction should be identified for the test categories X/O and O/X (Figure 4A). However, after performing the pre-planned, Matlab-based analysis, and then breaking the blinding code, no statistically significant effects could be identified for test categories X/O and O/X (compare Figures 4A,B).

In summary, the comparison of the *prediction* for true-positive results (see the full bars in Figure 4A) with the *actual* results as obtained with the true-experimental conditions X/O and O/X (see Figure 4B) found no matches between predicted and actual outcome measures for these test categories. That is, neither of the two pre-specified target criteria, namely, (1) the specific test category for which the true-positive effect was predicted to occur nor (2) the specific direction of the predicted effect (a decrease and an increase), found a statistically significant match with any of the actual results (i.e., for X/O or O/X) as measured with the true-experiment (compare Figures 4A,B). Therefore, the true-positive rate of a match between predicted true-positive results and actual true-positive results was 0% (0 of 2). Obviously, this finding was unable to reject any of the two null hypotheses for true-experimental test categories X/O and O/X (see H_0_-true-X/O and H_0_-true-O/X in Table 1). Next, the pre-specified prediction for the two true-positive results will be compared with the actual results as obtained with the *sham*-experiment (see Section “Prediction Versus Actual Result in the Sham-Experiment”).

### Prediction Versus Actual Result in the Sham-Experiment

As a reminder, the chief purpose of the sham-experiment is to test for the possible appearance of false-positive effects, which, if they occurred in the true-experiment, would be indistinguishable from true-positive effects. Given the prediction of two true-positive results in the true-experiment, only two possible false-positive results, namely, only those *duplicating key features* of the predicted true-positive results, could be misidentified as true-positive results. Specifically, a false-positive effect could be mistaken for a true-positive effect (1) if it occurred either in test category X_S_/O_S_ (X/O) or in test category O_S_/X_S_ (O/X) and (2) if it occurred in the predicted effect direction for the respective true-positive effect. The key point is the following: while eight different, statistically significant positive measurement results are – in principle – possible in the sham-experiment, in view of the overall effects prediction, only two of these eight possible outcomes could represent false-positive observer effects duplicating the predicted key features of true-positive observer effects.

**Figure 5:**
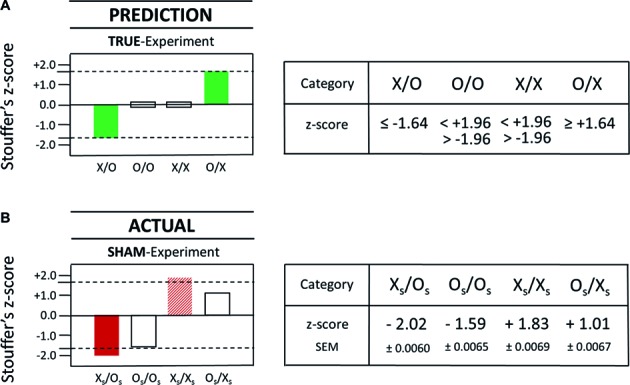
Comparison of the actual results from the sham-experiment with the pre-specified outcome predictions for the true-experiment. The statistical *prediction* for test categories X/O, O/O, X/X, and O/X is displayed as a graphical (left) and a numerical (right) representation in **(A)**. The *actual* statistical results that were obtained with the *sham*-experiment are displayed as graphical (left) and numerical (right) representations in **(B)**. Specifically, the results of the commissioned replication study are shown for the four tested null hypotheses, i.e., H_0_-sham-X_S_/O_S_, H_0_-sham-O_S_/O_S_, H_0_-sham-X_S_/X_S_, and H_0_-sham-O_S_/X_S_ (compare Table 1). Again, as was already described in Figure 4, each Stouffer *z*-score summarizes 1,250 *z*-scores calculated for each test pair which comprised a given test category, and the *dashed* lines represent the *z*-score magnitude at ±1.64, which is the cutoff for statistical significance (one-tailed). As before (see Figure 4B), the total number of dedicated test trials is identical for each test category, i.e., *N* = 1,250 for X_S_/O_S_, O_S_/O_S_, X_S_/X_S_, and O_S_/X_S_, yielding a total of 5,000 test trials for the complete sham-experiment. Importantly, *none* of the test trials used in the testing of one null hypothesis (e.g., H_0_-sham-X_S_/O_S_) was used again for the testing of another null hypothesis (e.g., H_0_-sham-X_S_/X_S_). Therefore, as was explained in Section “Statistical Interpretation of True- and Sham-Experiments,” a statistical correction for multiple testing is *not* applicable in the strictly *predictive* AMP-based research design.

What follows is a direct comparison, therefore, of the pre-specified *prediction* for a true-positive observer effect in the true-experiment (Figure 5A) with the *actual* result as obtained with the sham-experiment (Figure 5B): A complete match was identified for both target criteria in one of the two sham-test categories mimicking test categories X/O and O/X. Specifically, the actual result in the sham-test category X_S_/O_S_ (see the left full bar in Figure 5B) matched precisely the outcome predictions for the X/O-test category in the true-experiment (see the left full bar in Figure 5A). That is, *each* of the two target criteria, namely, (1) the specific test category and (2) the specific direction of the effect (a decrease) were matched by the actual result as observed with the sham-experiment X_S_/O_S_ (compare the left full bars in Figures 5A,B).

For explanation, the sham-experiment here seeks to identify potential false-positive effects, which, if they had manifested in the true-experiment for both (1) the predicted test category, and (2) the predicted effect direction, then they would be indistinguishable in appearance from true-positive effects (see also Section “Statistical Interpretation of True- and Sham-Experiments”). As a consequence, therefore, any positive results in test categories O_S_/O_S_ and X_S_/X_S_, or any positive results in the *non*-predicted effect direction for test categories X_S_/O_S_ or O_S_/X_S_, cannot – therefore – be mistaken for the *predicted* true-positive observer effect (compare Figures 5A,B).

The findings with the (meta-experimental) sham-experiment (compare Figures 5A,B) provide empirical evidence against the assumption that the measurement technique used by Radin et al. (2012) is *exclusively* specific for X, i.e., specific for the conscious state “concentrate” of a test subject (see Table 1); instead, there exists a significant sensitivity for (false-positive) factors or (confounding) influences other than observer consciousness. The probability that the false-positive effect in the pre-specified test category X_S_/O_S_ (see Figure 5B) was a mere chance event, i.e., due to random statistical fluctuations, was *p* = 0.021 (*σ* = −2.02; *N* = 1,250). For a more detailed analysis of this false-positive effect, consult Section “Percent Effect Size of the False-Positive Observer Effect,” where an overview is provided of all 1,250 individual X_S_/O_S_ ratio values, which were collected during 250 test sessions (i.e., five ratio values per single test session) over a time period of several months.

For completeness, the results for the second predicted true-positive effect, i.e., for the O/X-test category (STC) in the true-experiment (see right full bar in Figures 4A, 5A) were not found to be matched by the actual results in either the true-experiment (O/X; see right open bar in Figure 4B) or the sham-experiment (O_S_/X_S_; see right open bar in Figure 5B). It is noteworthy also that an *apparent* effect increase was associated with the sham-test category X_S_/X_S_ (see the *striped* bar in Figure 5B), which approached statistical significance at *σ* = +1.83 (*p* = 0.067; Figure 5B). This result was *not* however statistically significant because a two-tailed cutoff at *σ* = ±1.96 was used since the pre-specified hypothesis did not make a prediction about the *directionality* of possible systematic class-A error (Section “Sham-Experiment: Counterfactual Meta-Experimentation”), thus requiring a two-tailed *t*-test for analysis. Any systematic bias or imbalance that might reveal itself in the performance of the SNC- and SPC-experiments will manifest in one direction only for the particular test category (for an explanation see Section “Three Additional Control Test Categories”). Therefore, in the conservative approach toward identifying possible artifacts, a future confirmatory experiment might next posit a directional hypothesis with a cutoff at *σ* = +1.64 or at *σ* = −1.64 for that test category.

In summary, the commissioned study found that the used measurement process itself – without the application of any X- or O-conditions, i.e., in the complete absence of the test subject-dependent conscious states “concentrate” or “relax” (see Table 1) – produced a statistically significant (false-positive) effect in exactly the test category for which one of the two true-positive observer effects was predicted to occur (compare Figures 5A,B). This finding rejects the null hypothesis for the sham-experimental test category X_S_/O_S_ and identifies the presence of statistically significant class-A error for that test category, i.e., systematic error in association with the measurement process itself (see Section “Sham-Experiment: Counterfactual Meta-Experimentation”). The false-positive detection rate, here defined as the matching rate between *predicted* true-positive results, on the one hand, and *actual* false-positive results, on the other hand, was determined to be 50% (1 of 2) in the commissioned replication study of the Radin DS-experiment.

This AMP-based finding demonstrates experimentally, by way of the stringent test with the sham-experiment, that a false-positive effect could be mistaken for a true-positive effect with the research design as employed in the Radin DS-experiment and as tested in this commissioned study (compare Figures 5A,B). Obviously, that possibility would have remained hidden from the investigator in the absence of performing the sham-experiment, which can either confirm or disconfirm an acceptable true-negative detection rate in association with sham-test categories X_S_/O_S_ and O_S_/X_S_. Given the above findings (Figure 5), a non-negligible probability, therefore, exists that the previously reported, true-positive observer effect in the Radin DS-experiment might – in truth – represent a false-positive effect.

### Percent Effect Size of the False-Positive Observer Effect

**Figure 6:**
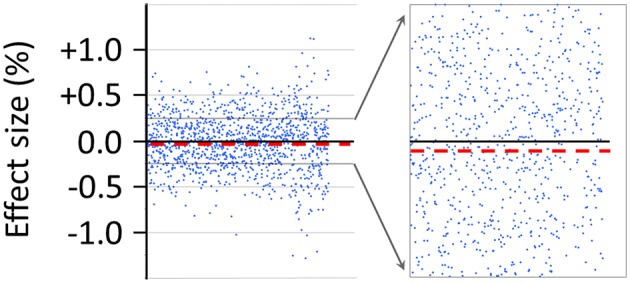
Effect size in percent of the false-positive observer effect as detected with the sham-experimental test category X_S_/O_S_ (compare Figure 5). The effect size marked by each dot in the graphs represents a measure of the difference between the means of two consecutive 30-s epochs (i.e., X_S_ and O_S_), as calculated by the pre-programmed Matlab script, and as expressed as the percentage of the mean of the first 30-s epoch (X_S_). In all, *N* = 1,250 ratio values are shown for the sham-experiment X_S_/O_S_ to the left of the figure. These data represent a total of about 20 h of recordings for the sham-experiment X_S_/O_S_ alone, which were obtained over a time course of several months using the AMP (see Section “Insertion of the AMP Into the Radin DS-Experiment”). The progression in time of collected recordings is from left to right. To the right of the figure is illustrated the magnification – in the range from ±0.25% – of the distribution of the ratio values (%). The *dashed* line in the figure marks the mean effect size (−0.0159%), which is a measure of the amount of SME in the form of *class-A error* as detected by the AMP-based X_S_/O_S_-test category. For the calculation of the effect size, see also the main text.

An estimate of the false-positive effect size in percent, which is associated with the sham-experimental test category X_S_/O_S_, will be described next. While this estimate was not a pre-planned outcome measure, the estimate was nevertheless calculated as an *exploratory* measure given that the 1,250 ratio values, including their sequential positions in time, were readily available as part of the pre-planned analysis with the original Matlab script (see Figure 6). As an exploratory measure, from these 1,250 individual ratio values, which were collected for X_S_/O_S_-test trials during the course of the whole study, was calculated an *absolute* mean difference (*d*_m_) between X_S_ (first 30-s epoch) and subsequent O_S_ (second 30-s epoch) as expressed as percentage of the mean of the first 30-s epoch (X_S_): *d*_m_ = 0.0159% (±0.0085 SEM).

The detailed view of the data in Figure 6 serves to illustrate the great challenge that presents itself in the Radin DS-experiment: To reliably detect evidence for ultra-weak effects on the order of 0.001%, upon using a measurement device that generates data scattering routinely in the range of about ±0.5% (see Figure 6), the experimenter needs to control rigorously for subtle confounding factors, some of which might *never* be identified and tracked, over the time course of many months of data collection. Importantly, lacking the technical ability to track *all* confounding factors, a systematic imbalance or bias in the measurement system could easily remain hidden – unless is performed the sham-experiment which reduces the risk of mistaking a false-positive effect due to hidden bias for a true-positive effect (compare Figures 5A,B). In summary, the experimenter is confronted in the Radin DS-experiment with a 1-to-500 difference between the expected effect size (%) as a potential function of observer consciousness and the degree of scattering (%) of ratio data points for individual test trials (see Figure 6).

The here described false-positive effect (*d*_m_ = 0.0159%) places an operational limit – after performing 250 test sessions – on both the specificity as well as sensitivity of the used instrument’s ability to detect potentially true-positive effects in the test categories X/O and O/X as a function of observer consciousness. For a near-perfectly balanced (i.e., sufficiently unbiased) physical measurement system, the difference ratios are predicted to be near-randomly distributed around the zero-line (see Figure 6). In the AMP-based replication study – as an indicator of SME – was observed a deviation or systematic off-set from the zero-line by −0.0159% for the Radin DS-experiment after recording 1,250 ratio values during 250 test sessions (see *dashed* line in Figure 6). For completeness, the percent effect size of *potential* class-A error in the X_S_/X_S_-test category (see Section “Prediction Versus Actual Result in the Sham-Experiment”) was 0.0118% ±0.0082 SEM (not shown). In summary, as shown in Figure 6, the size of the measurement bias or imbalance in the Radin DS-experiment due to class-A error can be about 10 times larger than the size of the claimed anomalous consciousness effect at 0.001% (Radin et al., 2016). For more discussion of this operational constraint see Section “The Percent Effect Size Is Comparable to That in Prior Work.”

The above-described findings suggest that the performance of the true-experiment alone, including of the SNC-based test category (O/O), would have been an insufficient strategy for detecting false-positive effects as an indicator of (hidden) bias in the Radin DS-experiment. It was the (meta-experimental) sham-experiment, which closely replicated all four true-experimental categories without test subjects (compare Figure 1B), that was capable of identifying the false-positive detection rate at 50%, indicating a measurement bias or SME in the form of class-A error (see Figures 5, 6). Critically, short of adopting the complete AMP, the measurement bias, e.g., due to an uncontrolled for sensitivity of the method to factors *other* than intentional observer consciousness, would have remained hidden. Consequently, lacking the *full* AMP-based approach, the possibility of misinterpreting a false-positive for a true-positive observer effect would have remained hidden also. A way forward toward identifying the possible source(s) of the false-positive effect in the Radin DS-experiment will be discussed in the subsequent Section “Discussion.”

## Discussion

The focus of the present discussion is the observation – by way of the confirmatory AMP-based approach – of the statistically significant (false-positive) effect in association with the sham-experiment in the absence of test subjects (see Figures 5, 6). Obviously, the identification of a significant effect in the *absence* of observer consciousness in the Radin DS-experiment would have to call for an alternative explanation other than an observer-based consciousness effect. Again, until the question concerning the true source of the false-positive effect can be answered, any claims are premature regarding the potential discovery of an anomalous quantum consciousness effect in the Radin DS-experiment (Radin et al., 2012). Given the working hypothesis by Radin et al. (2012) that the *specific* state of test-subject consciousness, i.e., the intentional state associated with the instruction “concentrate” (see Table 1), is the *only* possible cause of the claimed anomalous effect (Radin et al., 2012, 2013, 2015, 2016), the finding of the commissioned replication study is deeply concerning that the false-positive detection rate reached 50% as determined with the sham-experiment (see Section “Prediction Versus Actual Result in the Sham-Experiment”). For a brief discussion of the inability to replicate the original findings of Radin et al. (2012), i.e., those claiming a “true-positive” consciousness-based effect, see Section “On the Failure to Replicate the Original Findings.”

### Four Reasons Calling for Skepticism

Short of identifying the specific source or cause that is responsible for the false-positive detection rate in the Radin experiment (see Section “Prediction Versus Actual Result in the Sham-Experiment”), any claimed true-positive, anomalous effect should be viewed with considerable doubt (e.g., Radin et al., 2012). Why is such a strong skeptical conclusion warranted here? A review of major reasons follows next, explaining why a skeptical position is recommended – at least at the present time – in relation to the widely discussed prior claims (Radin et al., 2012, 2013, 2015, 2016).

#### The Effect Is Observed in the Absence of Test Subjects

The cause of the false-positive finding remains unknown until now. Critically, that very same cause – whatever it may be – could be the source also, until proven otherwise, of the claimed psycho-physical effect in the Radin DS-experiment. Again, of all the previously published work, which had reported an anomalous observer effect in the Radin DS-experiment, *none* has – obviously – adopted the new meta-experimental strategy which was implemented in the commissioned replication study. Again, the AMP is based upon (1) the joint application of randomly assigned SNCs, SPCs, and STCs (see Section “Three Additional Control Test Categories”), in combination with (2) a full set of sham-experiments which reproduce these test categories, however, in the absence of test subjects. Never before has the Radin DS-experiment been subjected to such demanding tests in the search for potential measurement bias. Importantly, it was the AMP-based sham-experiment (see Section “Sham-Experiment: Counterfactual Meta-Experimentation”) that accomplished the quantification of the false-positive detection rate in relation to the DS-device and the pre-specified statistical analysis (see Figure 5). Finally, this commissioned study is the first *conceptual* replication attempt of the original Radin DS-experiment (Radin et al., 2012) which implemented a fully pre-specified research design in agreement with blinded and strictly *confirmatory*, instead of *exploratory*, research practices (see Table 3).

**Table 3:** Overview for the Radin DS-experiment of the available publications, including the present work, illustrating the combinations of (1) direct *versus* conceptual replication attempts, with (2) confirmatory *versus* exploratory study designs.

	Confirmatory study design	Exploratory study design
Direct replication	Radin et al., 2012	
Conceptual replication	The present work	Radin et al., 2013, 2015, 2016

Note that one of the six experiments reported in the original study (Radin et al., 2012) did adopt a confirmatory study design. For an explanation of the technical terms, see Section “Materials and Methods.”

#### The Statistical Significance Is Comparable to That in Prior Work

The results of the commissioned replication study have not merely raised the possibility of artifacts but have demonstrated an actual artifact – in this case – in association with the DS-experimental system. Again, even when the Radin DS-experiment was performed with great care – as was the case with the commissioned study – a false-positive detection rate of 50% was observed (see Figure 5). Importantly, the degree of statistical significance of the here identified false-positive effect is on the same order as was reported for the apparently true-positive effect in the original Radin DS-experiment, namely, a *z*-score on the order of two (Radin et al., 2012). That is, Radin and co-workers have routinely interpreted *z*-scores on the order of two sigma as representing significant evidence for an anomalous consciousness effect. For example, they have carried out a combined statistical analysis of the first four (of the six) separate experiments that made up the complete original study: A *z*-score of *σ* = −2.17 was calculated for the total, combined result across the four experimental sets, which consisted of 121 experimental sessions (Radin et al., 2012); for comparison, a *z*-score of *σ* = −2.02 was calculated for the total data set in the sham-experiment X_S_/O_S_ in the present study (see Figure 5).

#### The Percent Effect Size Is Comparable to That in Prior Work

The size in percent of the here identified false-positive effect, i.e., *d*_m_ = 0.0159% (X_S_/O_S_; see Section “Percent Effect Size of the False-Positive Observer Effect”), is within an-order-of-magnitude of the estimated effect size (0.001%) for the anomalous consciousness effect which has before been reported by Radin et al. (2016). Therefore, regarding the percent effect *size*, at least, of the false-positive effect, an alternative, *non*-consciousness-based explanation might fully account for – until proven otherwise – the previously announced anomalous effect (Radin et al., 2012, 2013, 2015, 2016). Importantly, the experimental detection of SMEs on the order of 0.01% places an operational limit on the sensitivity as well as specificity of the employed DS-system (for details see Section “Percent Effect Size of the False-Positive Observer Effect”). To be sure, should one wish to measure larger effect sizes, i.e., weak effects on the order of 0.1–1.0%, then the Radin DS-experiment could potentially be suitable for such a task, because the detected degree of class-A error is significantly lower by comparison. However, for claimed ultra-weak effect sizes which are 100 to 1,000 times smaller than that, such as potential effects on the order of 0.001% (Radin et al., 2016), the Radin DS-experiment might be prone to falsely identifying an intrinsic imbalance, or a non-specific detection effect, for a true-positive finding (compare Figure 6).

#### The Prior “Robot Experiments” Do Not Equal the Advanced Meta-Experimental Protocol-Based Control Design

The control experiments described in the previously published work (e.g., Radin et al., 2012, 2016) with so-called “robot” participants, i.e., performing robot, or sham-type, experiments, do not equal – in crucial respects – the here adopted sham-experimental tests as part of the AMP-based research strategy. For example, the robot control experiments by Radin et al. (2012, 2016) were *not* done systematically, i.e., in a strictly *paired* manner, as was, by contrast, the case with the sham-experiments as adopted in the commissioned replication study (see Figures 1, 3). Importantly, it is impossible to confirm whether the robot control experiments in the *prior* conceptual replication attempts of the Radin DS-experiment (Radin et al., 2013, 2015, 2016), which were neither performed in the confirmatory mode nor – as was already suggested – in a strictly paired manner, did – in fact – have sufficient power to rule out the presence of hidden sensitivities of the test method to ultra-weak influences other than observer consciousness. Importantly, based on the results obtained with the present replication study, the possibility cannot be excluded that the prior Radin DS-experiments have produced measurement outcomes that could either be false-positive or – in the case of the robot control experiments – “false-negative.” In this specific context, the meaning of the term “false-negative” is *not* the one in the usual sense of “type-2 error” (see Table 2), but one in the sense of falsely indicating the absence of measurement bias by the robot or sham-type experiment. This possibility will be referred to as “type-FNC error,” which indicates a “false-negative control,” i.e., the *false* indication of the specificity of the used detection method for the tested intervention (Walleczek, in preparation). The implementation of the complete and confirmatory AMP-based research design can greatly reduce the risk of type-FNC error also. In conclusion, the previous control experiments can be no substitute for the complete adoption of the AMP, because the capacity of these sham-type or robot control experiments to *empirically* assess the presence of potential systematic error is diminished greatly compared to the power of the confirmatory and strictly predictive AMP-based research strategy (see Section “Advanced Meta-Experimental Protocol”). Recent evidence from an independent re-analysis of sham-type control data from *prior* Radin DS-experiments supports this view also: Tremblay (2019) reported that “part of the control data is also found anomalous,” and that this “undermines the anomalies found in the human data, and weakens possible conclusions to be drawn from this dataset.”

### On the Failure to Replicate the Original Findings

The question of why no intentional observer effect could be identified in the commissioned replication study will not be discussed in great detail here (see Figure 4). Suffice it to say that the lack of positive findings does not of course – by itself – invalidate the studies that had reported apparently positive effects until now (Radin et al., 2012, 2013, 2015, 2016). That is, under the (optimistic) assumption that the *original* Radin DS-experiment had – in fact – identified a genuine observer consciousness effect, there could be many possible reasons for why the present study has failed to replicate the original results (Radin et al., 2012). For example, the *failure* to replicate findings in anomalous cognition, or parapsychology in general, was proposed to be a *predicted* feature of anomalous consciousness effects in the laboratory (Walach et al., 2014). Briefly, these authors recommended to “… never repeat experiments exactly the same way, but always change some parameters,” because the failure to replicate “… only arises with exact replications.” It should be pointed out, however, that in the specific case of the Radin DS-experiment, *none* of the different follow-up studies, including the *present* confirmatory replication study also, represented exact or direct replications, but they all represented *conceptual* replications – always with variations in the used measurement and/or analytic parameters (compare Table 3). At present, it remains unknown, according to the above proposal, how large the experimental differences should be – between an original study and subsequent replication attempts – to be able to reproduce – at least in a statistical sense – the *general* anomalous finding (e.g., Radin et al., 2012, 2013, 2015, 2016).

Additional, less controversial, explanations exist also for the failure to replicate the original effect in the present study. For example, the claimed observer-consciousness effect might depend on subtle environmental factors and cues, or internal, psychological factors as well. The latter might include factors related to psychological feedback, mental pre-conditioning, general states of awareness, or even psycho-physical well-being, in association with test subjects. In fact, a form of real-time psychological feedback was included in most, but not all, experiments of the original study (Radin et al., 2012); however, none of the experiments in the commissioned replication study included psychological feedback (the reasons for this omission by Dean Radin, the researcher who was commissioned to replicate the original study, are not entirely clear; compare Section “Materials and Methods”). In any case, for the present analysis, the crucial point is the following: There does *not* exist any reason for assuming that the lack of psychological feedback in the commissioned replication study could be the source of the statistically significant false-positive effect that was identified in the *absence* of test subjects (see Figures 5, 6).

### In Search of an Explanation for False-Positive Observer Effect Detection

Two different options regarding SME might explain the false-positive detection rate in the Radin DS-experiment (Walleczek, in preparation): (1) SME associated with the experimental performance and raw data collection or (2) SME associated with the (pre)processing of the raw data and the statistical analysis; a combination of the two options might also be possible. Note that the possible presence of class-A and/or class-B error (see Section “Sham-Experiment: Counterfactual Meta-Experimentation”) might be associated with either of the two options. The first option is called *systematic experimental error* and it suggests that the employed measurement technique and/or data-collection method is (weakly) unbalanced or biased during the performance of the experiment. For example, the detection method may manifest a sensitivity to (as-yet) unknown physical factors which are beyond the ability of the particular method to reveal, track, and identify (compare Section “Advanced Meta-Experimental Protocol”). The second option is called *systematic statistical error* and this one suggests that not the experimental performance but the subsequent data processing routines themselves, such as normalization, outlier removal, detrending, smoothing, etc., may introduce unintended imbalances and create the (false-positive) appearance of an anomalous consciousness effect when in fact there is none (see also Section “Implementing the Confirmatory Research Design”). Importantly, systematic errors associated with either option (1) or option (2) can be part of what has been termed the SME-loophole in Section “Introduction.”

An example of the failure to recognize the presence of a false-positive effect due to SME is the before-mentioned model of the biased or unbalanced roulette wheel in a casino (for details see Section “Introduction”): In the case of the roulette wheel, the failure to close the SME-loophole might lead an observer of the game to believe that an unusually successful player was, for example, either incredibly lucky, or even had “psychic abilities” in influencing the spinning wheel, when in truth there was an uncontrolled physical bias in the system, which the player happened to exploit – either knowingly or unknowingly. In either case, a spectator at the roulette table would be very impressed, when – in truth – a hidden physical bias was responsible for increasing the chances of winning ever so slightly, but nevertheless significantly. Similarly, in the case of the prior Radin DS-experiments (Radin et al., 2012, 2013, 2015, 2016), the failure to close the SME-loophole is likely to have led to the mistaken identification of false-positive for true-positive results. A subsequent article will specifically deal with the risk of producing false-positive conclusions with the Radin DS-experiment based upon option (2), i.e., systematic statistical errors, for example, due to the lack of pre-specified replication designs in the prior Radin DS-experiments (von Stillfried and Walleczek, in preparation).

## Conclusion

The finding that the Radin DS-experiment can produce a statistically significant effect in the *absence* of test subjects for a *predicted* test category casts doubt on the scientific validity of the claimed (true)-positive effect which has been reported before (Radin et al., 2012, 2013, 2015, 2016). Specifically, the determination of the false-positive detection rate at 50% has strong implications also for future independent replication attempts of the Radin DS-experiment (see Figures 5, 6). Lacking the adoption of the complete AMP (see Section “Advanced Meta-Experimental Protocol”), it cannot be reasonably assessed whether an apparent positive effect, if indeed it is found, is not – in truth – a false-positive effect after all. For example, the detection of the *false-positive* observer effect could appear to be replicable across independent studies even. Therefore, a so-called “successful” replication attempt need *not* imply the confirmation of a “true-positive” observer effect. In analogy to the secretly unbalanced roulette wheel (see Section “Introduction”), the conduct of a *new* Radin DS-experimental series might be like moving to another roulette table in the casino, with the spinning wheel being unbalanced in a *new* and (as-yet) uncontrolled for manner; this as a function of hidden and – for a given time frame – systematic imbalances in the physical system in combination with unknown, ultra-weak influences from the surrounding environment which impinge on the system in subtle, and sometimes systematic, ways (compare Figure 6). Therefore, lacking the stringent meta-experimental approach outlined here, even independent replication studies could be at risk of producing false-positive conclusions. This is a particularly great concern when adopting an *exploratory* study design only (see Table 3), i.e., when no special effort is made to confirm an acceptable true-negative detection rate with a *confirmatory* AMP-based replication design for *all* test conditions and test categories.

Consequently, extensive new work would need to be carried out, especially with (1) fully pre-specified conditions and analyses as well as (2) the routine insertion of the complete AMP into the experimental design, before this all-important question could possibly be answered in the negative: Might the astonishing claims regarding the previously published Radin DS-experiments have come about due to the misidentification of false-positive for true-positive results? Put differently, since the false-positive rate of detection was 50% in the so far most carefully designed Radin DS-experiment, i.e., the commissioned replication study, and since – in the prior studies – the efforts have been insufficient to eliminate the possibility of type-1 error (see Section “Four Reasons Calling for Skepticism”), the question of the true origins of the claimed anomalous effect is of pivotal importance and – unfortunately – that question remains unresolved at present (see Section “In Search of an Explanation for False-Positive Observer Effect Detection”).

For the future, our recommendation is for researchers to discontinue the *exploratory* research approach toward the Radin DS-experiment (see Table 3) and to start implementing the methods and protocols that are consistent with strictly *confirmatory* research practices and designs (e.g., Simmons et al., 2011; Wagenmakers et al., 2012; Munafò et al., 2017; von Stillfried and Walleczek, in preparation); only confirmatory research designs might put to rest the long-standing questions about the possibility of type-1 error as part of any conceptual replication attempt relating to the purported consciousness effect. Recommended is the performance of a series of confirmatory replication studies of the Radin DS-experiment, whether they are conceptual or direct replication attempts, with all of them implementing the fully predictive, AMP-based research strategy (see Section “Advanced Meta-Experimental Protocol”). The results gathered by conducting a short series of confirmatory studies might direct researchers toward the identification of the true source of the false-positive effect (see Figures 5, 6). Until the discovery of the true origins of the false-positive detection rate in the Radin DS-experiment, skepticism should replace optimism concerning the radical claim that an anomalous quantum consciousness effect has been observed in a controlled laboratory setting.

## Data Availability

All datasets generated for this study are included in the manuscript and/or the supplementary files.

## Ethics Statement

The Institutional Review Board of the Institute of Noetic Sciences approved the study and requested of each study participant to sign an informed consent form, which stated that participation in the study was voluntary, and could be discontinued at any time.

## Author Contributions

JW contributed to the conception and the design of the study. NS contributed to the organization of the data base and the statistical analysis. Both authors contributed to writing the manuscript and have approved the submitted version.

### Conflict of Interest Statement

The authors declare that the research was conducted in the absence of any commercial or financial relationships that could be construed as a potential conflict of interest. In particular, the relationship between JW and the funding organization was disclosed in the article.

## Footnotes

^1^www.ions.org

^2^www.phenoscience.com
